# Supplementary material for: Importation of Alpha and Delta variants during the SARS-CoV-2 epidemic in Switzerland: Phylogenetic analysis and intervention scenarios
Source: PLoS Pathog. 2023 Aug 10;19(8):e1011553. doi: 10.1371/journal.ppat.1011553 (PMC10443857; doi:10.1371/journal.ppat.1011553)
Supplement: S1 File — (DOCX) [file ppat.1011553.s001.docx]

Supplementary material from manuscript entitled: **Importation of Alpha and Delta variants during the SARS-CoV-2 epidemic in Switzerland: phylogenetic analysis and intervention scenarios**

**Authors**: Martina L Reichmuth^1*+^, Emma B Hodcroft^1,2,3+^, Christian L Althaus^1,3^

**Affiliation**: ^1^ Institute of Social and Preventive Medicine, University of Bern, Bern, Switzerland; ^2^ Swiss Institute of Bioinformatics, Lausanne, Switzerland; ^3^ Multidisciplinary Center for Infectious Diseases, University of Bern, Bern, Switzerland

**Correspondence:** *martina.reichmuth@unibe.ch

^+^These two authors contributed equally to this work

**Supporting information**

- Method in S1 File
- Table A in S1 File
- Fig D-I in S1 File
- S1 Table (separate pdf file)
- S2 Table (separate machine-readable file)

**Method**

**Creating the Phylogenies**

**Selecting Swiss Sequences**

All Swiss Alpha and Delta SARS-CoV-2 sequences prior to 31 March 2021 and 31 July 2021, respectively, were selected using code from CoVariants.org and in-house scripts available at<https://github.com/emmahodcroft/Intros-CH-AlphaDelta>. These cutoff dates reflect when the variants reached 90% of all sequenced SARS-CoV-2 cases prevalence in Switzerland. These sequences were our ‘**focal set**’ and all were included in the analysis, though some were excluded in the phylogenetic pipeline for not passing quality control (QC) metrics. We started with 8,083 Alpha and 5,232 Delta sequences and 7,988 and 5,210 passed QC and were included in the final analysis.

**Selecting Contextual Sequences**

In order to detect introductions, we aimed to look at transitions on the phylogeny from ‘non-Swiss’ to ‘Swiss’ SARS-CoV-2 sequences; thus, we wanted to include ‘**context sequences**’ that were as closely related to our focal set as possible to maximize the probability of correctly separating Swiss sequences that could be separate introductions.

The Nextstrain ncov pipeline has built-in functionality that allows for the selection of background sequences by their ‘proximity’ (genetic distance) to the focal set. The relevant scripts can be seen at<https://github.com/emmahodcroft/ncov_2021/tree/random_context_reduce> in the ‘scripts’ folder. The `get_distance_to_focal_set.py` script (the `proximity_score` rule in ncov) generates a matrix of genetic distances between every sequence in the focal set and all of the potential context sequences (here, the rest of the dataset). Every potential context sequence is assigned to its closest focal set sequence, resulting in every focal sequence having a set of context sequences associated with it.

The `priorities.py` script builds on this by going through every focal sequence and its associated set, and assigning a score to each associated sequence, based on the genetic distance, but lowered by the count of ambiguous and ‘N’ bases. The associated sequence list is first put in random order and then sorted by scores, highest (closest genetic distance and highest quality sequences; around -1) to lowest (for example -100). Scores are then further fractionally lowered by their position in the resulting list. This fractional lowering, or ‘crowding penalty,’ ensures that a focal sequence with many, close matches does not have all their associated sequences chosen at the expense of other focal sequences. For example, to ensure the algorithm later selects the top best-scoring sequence match for sequence X over taking the 10th best-scoring sequence match for sequence Y, all else being equal.

These scores are passed into the `filter` step of ncov which uses the priority scores to select the specified number of sequences requested (here 10,000) and generate a set of non-Swiss sequences closely related to the focal (Swiss) set. As the context set may include sequences later excluded for QC reasons (ex: over/under divergence due to wrong date), the *final* context set is usually slightly smaller than 10,000. For our builds we *initially* selected 10,010 sequences each for Alpha and Delta.

**Selecting Random Background**

All ncov-based phylogenies are rooted by Wuhan/Hu-1/2019, and from experience the phylogenetic algorithms resolve more readily when there are a minimal number of sequences from January 2020 through the cutoff date of the run to provide a ‘backbone’ to the phylogeny. Thus, for each build 200 sequences were randomly selected, equally distributed by month between January 2020 and the cutoff date. Since the number of months may not evenly divide into 200, sequences may be selected both as random background and as contextual background, and sequences may be later excluded for QC reasons (as above), the final number of sequences is usually slightly lower. In our builds 187 random background sequences for Alpha and 171 for Delta were initially selected.

For the Alpha and Delta builds the total number of both contextual and random background sequences in the final analysis build was 10,063 for Alpha and 10,120 for Delta.

**Modifying Sequence Selection for the Reruns**

We can only detect introductions by looking at transitions from non-Swiss to Swiss sequences in our phylogenies, meaning our inference is dependent on non-Swiss sequences. We wanted to investigate how the level of sequencing outside of Switzerland may have impacted our analysis, alongside exploring variation in phylogenetic reconstruction. Thus, we created 10 reruns of the Alpha & Delta analyses, with each rerun excluding 50% of the available non-Swiss sequences.

For computational efficiency, the proximity genetic distances between all background sequences and the focal set were calculated only once (for Alpha and Delta each) and used for each re-run (since this never changes). However, during the priority step to convert these proximities into priorities, 50% of the sequences were randomly scored down by -10,000 (well below the lowest possible score normally), ensuring they are never selected for inclusion in the context set. Thus, only 50% of the possible context sequences were available for inclusion, simulating a situation where there was approximately 50% less sequencing outside of Switzerland.

**Collapsing Phylogenies & Inferring Introductions**

**Collapsing Phylogenies**

Since we are primarily interested in the transitions from non-Swiss to Swiss sequences on the phylogeny and never interested in further diversification within a single country, we aimed to simplify the phylogeny to highlight only these transitions, as described previously in Hodcroft et al. 2021.

This was done by recursively collapsing subtrees with sequences only from one country until a mixed-country polytomy was reached, after which collapsing stopped. In Fig A below, a simple scenario is shown with steps from left to right. Red represents a node with only sequences from country X, pink sequences are from country Y, and blue are sequences from Switzerland. As shown, all nodes that contain children from just one country are collapsed into polytomies recursively until a polytomy with mixed countries is reached. This can then be represented as a ‘pie’ (last step) showing the fraction of sequences from each country that make up this polytomy.


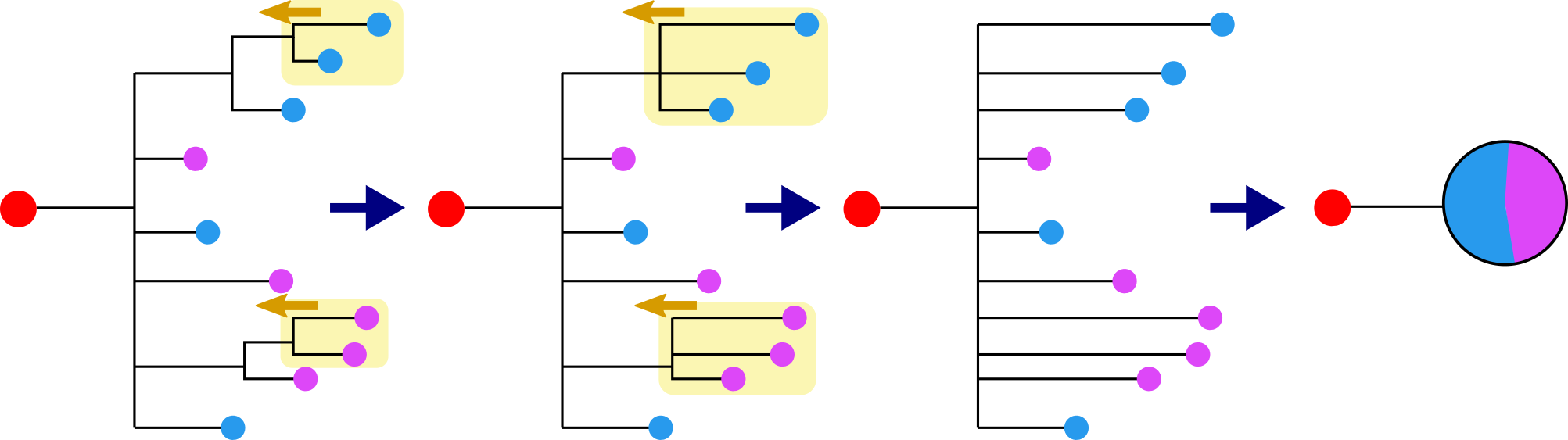


**Fig A.** Steps go from left to right as indicated by blue arrows. Collapsing a phylogeny recursively so that sub-trees with sequences from only one country are collapsed into a multi-country polytomy, and can be represented as ‘pies’ showing the fraction of sequences from each country. Here red is country X, pink country Y, and blue is Switzerland.

In a more complicated example in Fig B, one subtree is already a polytomy of mixed countries, thus cannot be collapsed further. Thus, this can be represented by two ‘pies,’ with the second being descended from the first.


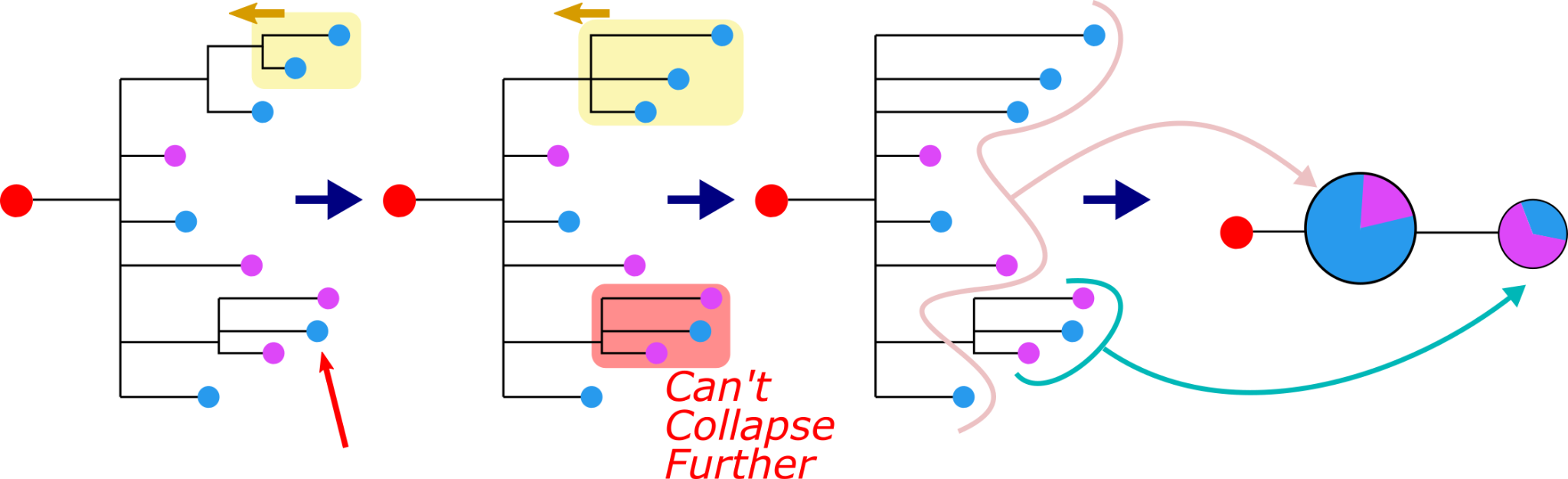


**Fig B.** Steps go from left to right as indicated by blue arrows. This phylogeny collapses like Fig A, but now has a sub-tree with sequences from mixed countries (indicated by the red arrow) which can’t be collapsed further, leading to two ‘pies’. As before, red is country X, pink is country Y, and blue is Switzerland.

**Inferring Introductions**

After collapsing we can traverse the phylogeny to identify ‘pies’ that contain a mix of Swiss and non-Swiss sequences and consider these as putative introductions. In a simple case, a node with no Swiss sequences (a mix of non-Swiss countries) leads to a node with Swiss sequences; this would always be classified as an introduction (Fig C - A, B, & C). However, one can also find nodes containing Swiss sequences descending from nodes containing Swiss sequences (Fig C - B, B1, B2). This could represent three scenarios: multiple separate introductions, parallel diversification in Switzerland and elsewhere (no introduction), and export from Switzerland to elsewhere (no introduction). Thus, we considered a liberal scenario where we consider each of these pies an introduction, and a conservative scenario where only the first node is considered an introduction, and all subsequent nodes are considered either parallel diversification or export from Switzerland.


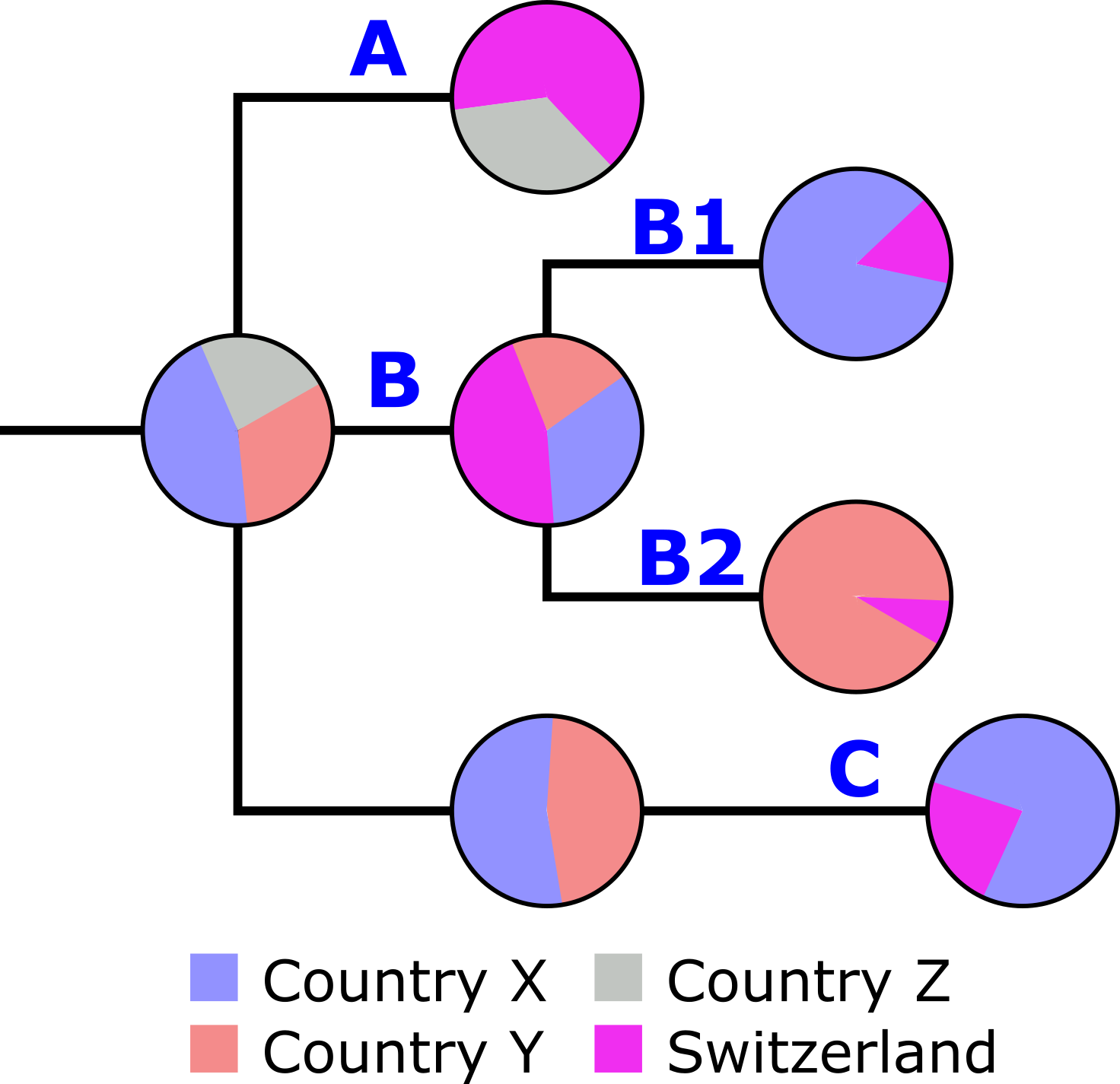


**Fig C.** A collapsed phylogeny represented as ‘pies’ of sequences from different countries. Swiss sequences are in pink. A, B, and C show straightforward scenarios where nodes with Swiss sequences descend from non-Swiss nodes; these are always counted as an introduction. B, B1, and B2 show one node that descends from non-Swiss nodes (always 1 introduction), but has 2 more nodes descending from it with Swiss sequences. In the ‘liberal scenario’ B, B1, and B2 would be considered 3 introductions. In the ‘conservative scenario’, B, B1, and B2 would be considered 1 introduction.**Table**

**Table A.** Estimated importation of VoCs and simulated impact on the SARS-CoV-2 epidemic in Switzerland. Abbreviation: VoC, variant of concern.

|  | **Alpha** | | **Delta** | |
| --- | --- | --- | --- | --- |
|  | **Liberal** | **Conservative** | **Liberal** | **Conservative** |
| **Estimated imports from the phylogeny** | 1,038 | 383 | 1,347 | 455 |
| **Simulation period** | 1 Oct 2020 - 1 May 2021 | | 1 Feb 2021 - 1 Sept 2021 | |
| **Total simulated reported cases** | 593,418 | 592,768 | 288,397 | 271,702 |
| **Number of simulated variant cases** | 97,116 (16%) | 70,898 (12%) | 110,596 (38%) | 87,861 (32%) |
| **Date by which 50% of cases are the variant (dominance)** | 05 Mar 2021 | 22 Mar 2021 | 30 Jun 2021 | 09 Jul 2021 |

**Figure**


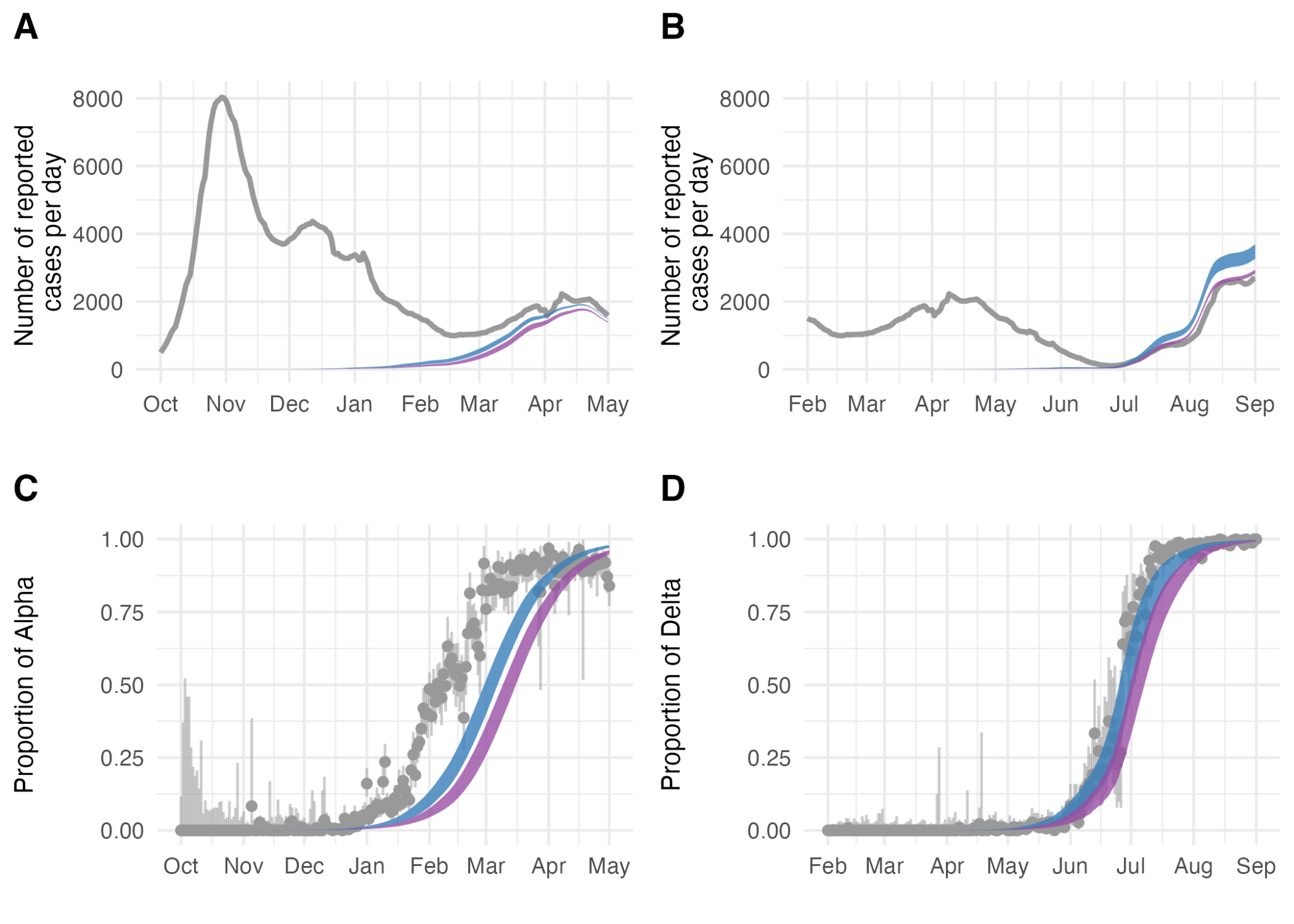
 **Fig D.** **Dynamics of Alpha and Delta importation to Switzerland.** This figure is similar to Figure 4, but the range was received from eleven simulations using different times of detection lags, namely 3-13 days in 1 day steps. This range was derived from du Plessis et al. (2021). A, B: Number of laboratory-confirmed SARS-CoV-2 cases per day (gray). The blue and purple area show the range of simulated number of VoC cases. C, D: The range of proportion of reported Alpha and Delta among all SARS-CoV-2 infections. Gray: Genomic surveillance data. Blue: Liberal approach. Purple: Conservative approach.


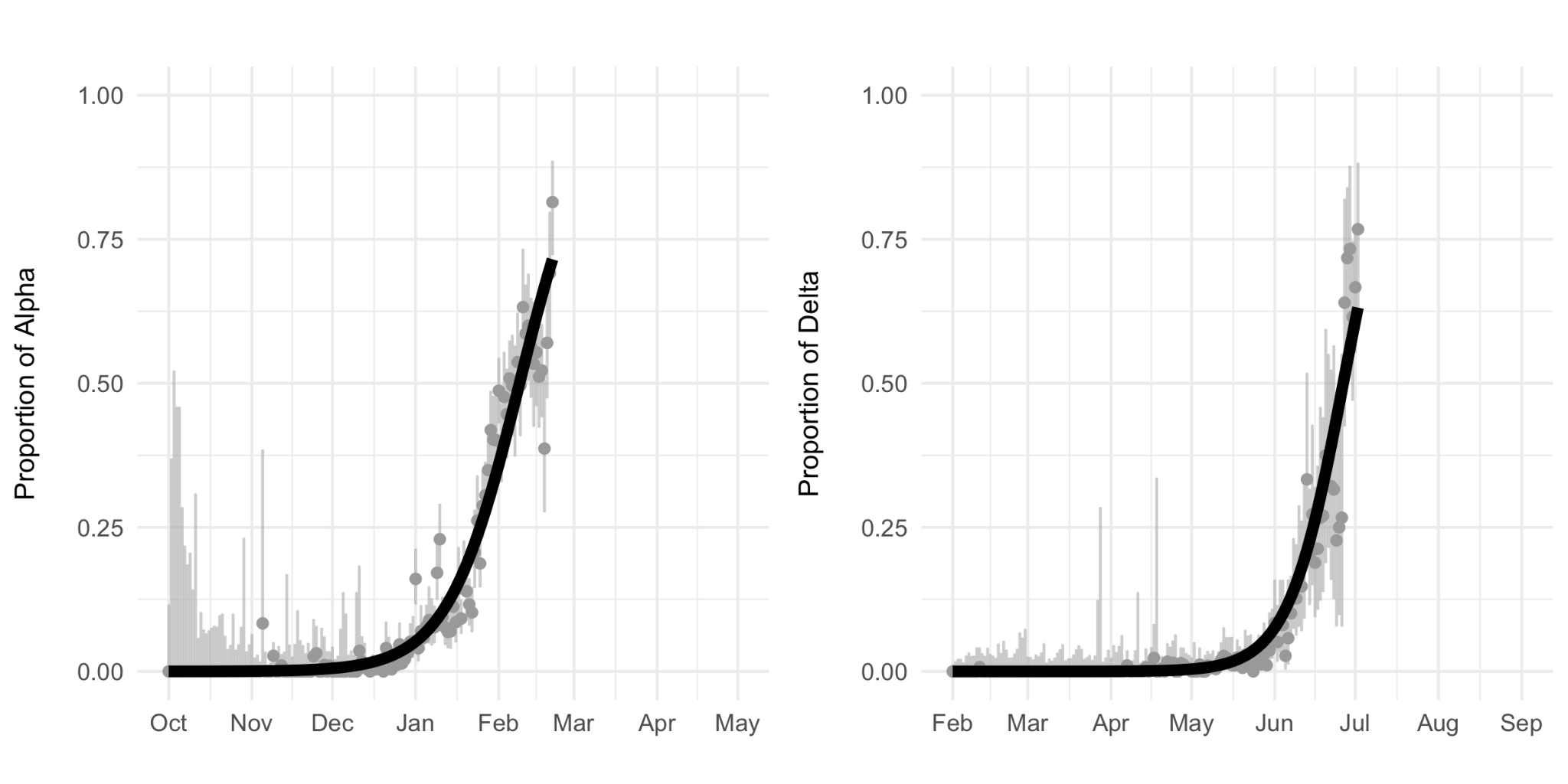
 **Fig E.** Fit of the logistic growth model to the proportion of SARS-CoV-2 VoCs. Gray dots: Genomic surveillance data with 95% binomial confidence interval. Black line: Model fit.

**
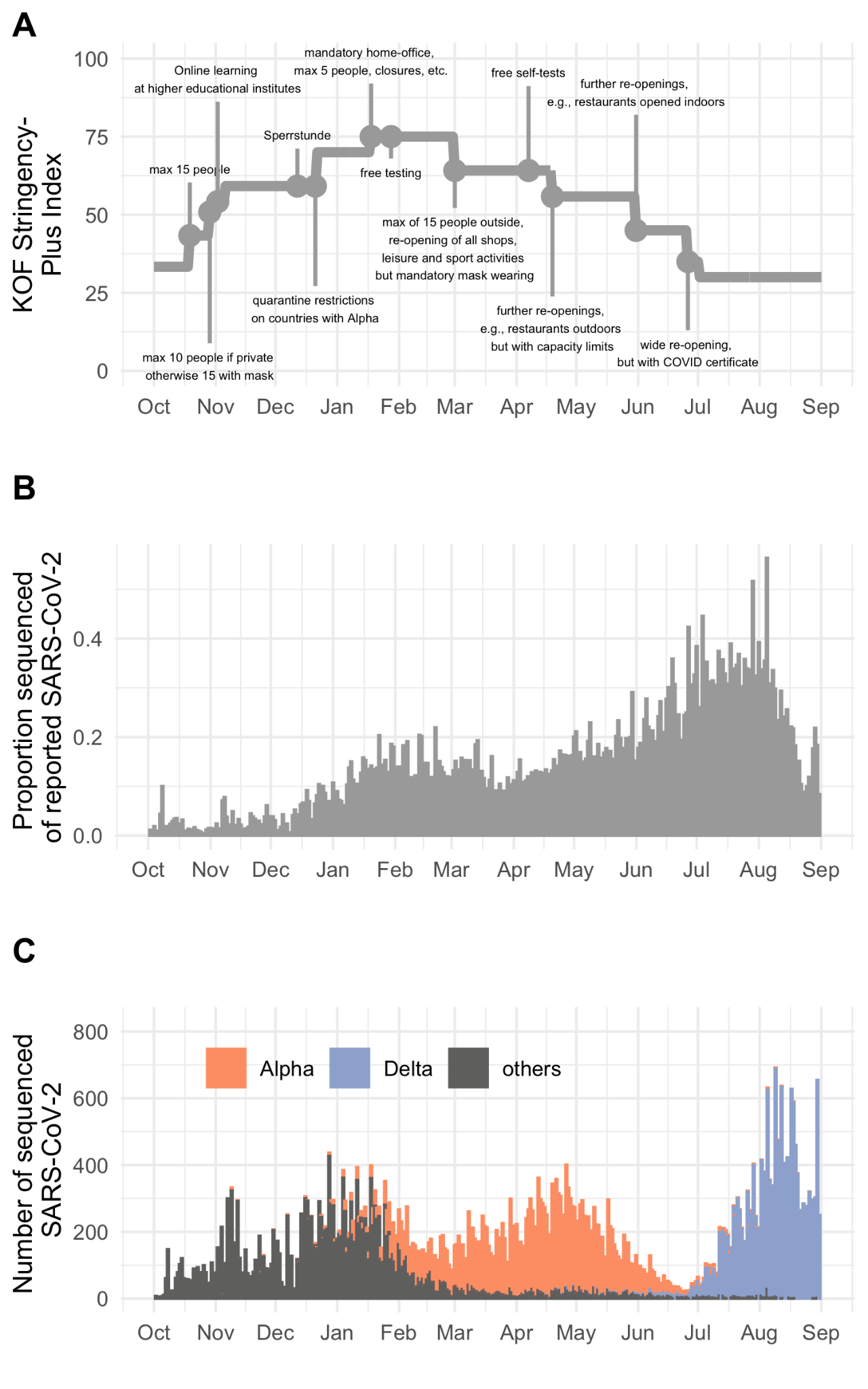
**

**Fig F.** Measures and genomic sequencing during the SARS-CoV-2 epidemic in Switzerland from October 2020 to September 2021. A: The [KOF stringency plus index](https://kof.ethz.ch/en/forecasts-and-indicators/indicators/kof-stringency-index.html) recorded the stringency of SARS-CoV-2 policy measured in Switzerland over time. The values range from 0 (= no measures) to 100 (= full lockdown). B: Proportion of reported SARS-CoV-2 cases that were sequenced. **C**: Number of sequenced SARS-CoV-2 cases.

**
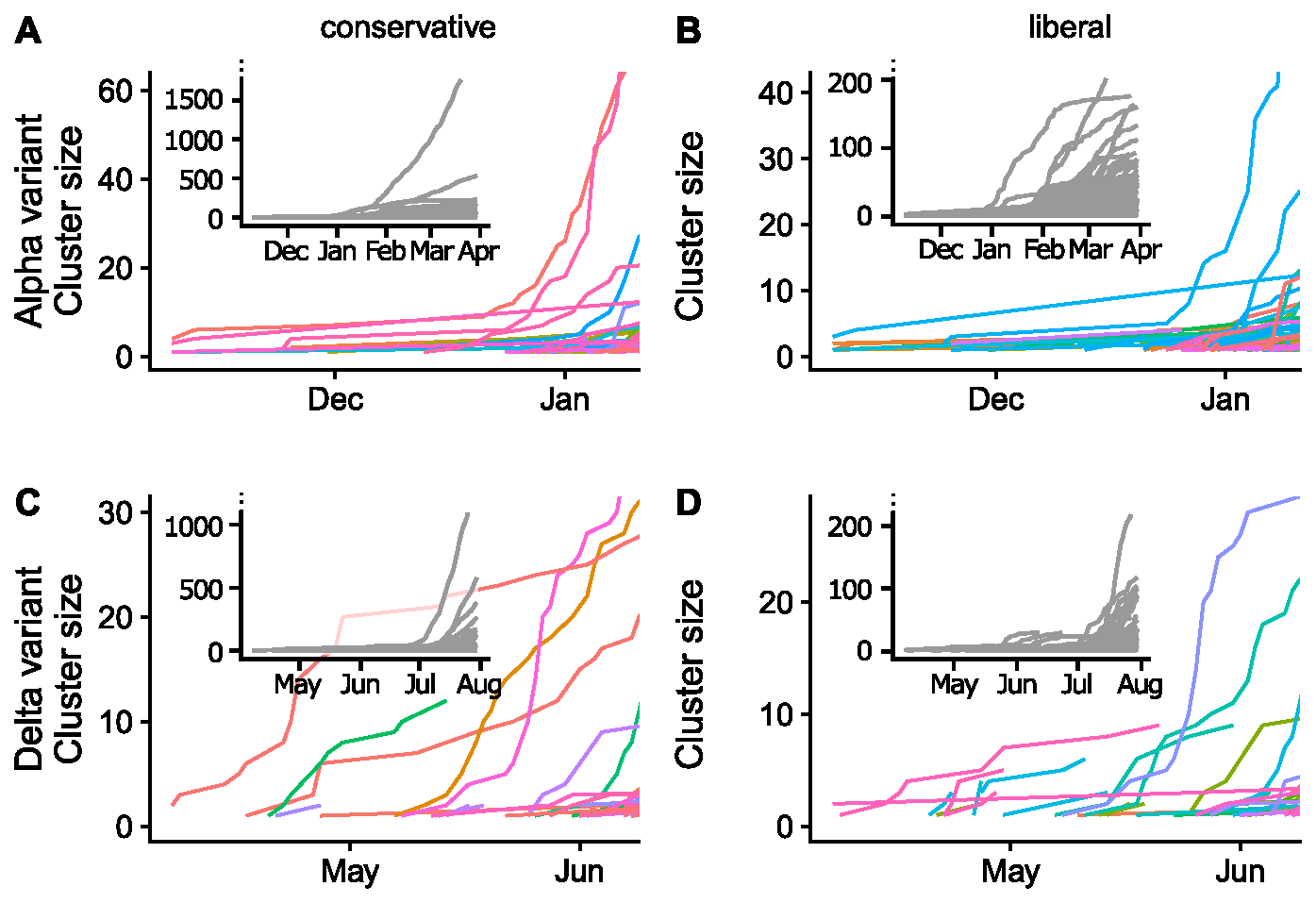
**

**Fig G.** The growth in Swiss sequences of the cluster that led to an import of a VoC. The plots show each cluster for the first two months as a line. The small zoomed plot shows the clusters over a longer time period. A: Alpha variant and conservative approach. B: Alpha variant and liberal approach. C: Delta variant and he conservative approach. D: Delta variant and liberal approach.


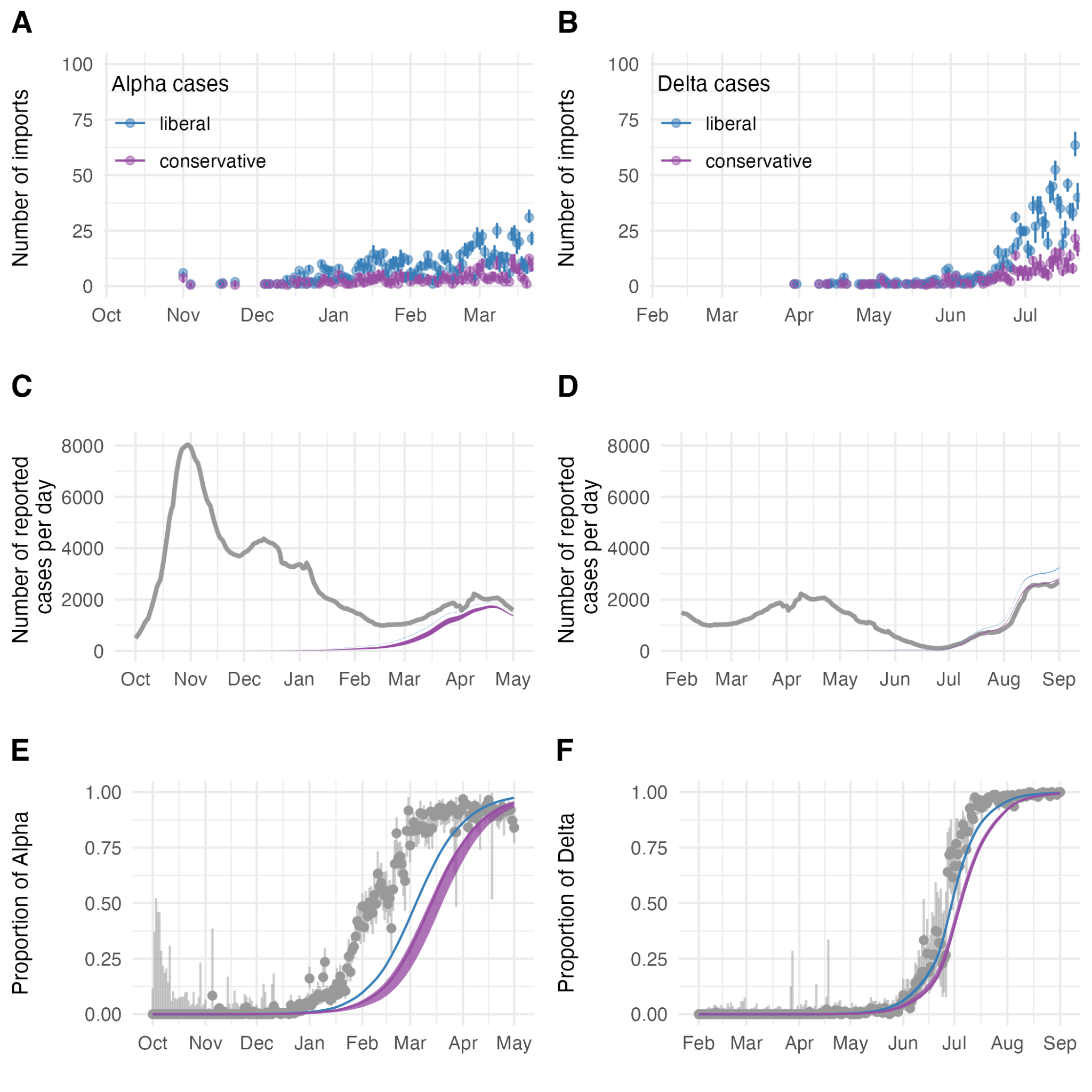


**Fig H.** **Dynamics of Alpha and Delta importation to Switzerland.** Range of the estimated number of imports and resulting model for ten reruns of the Alpha and Delta analysis, where the number of possible context sequences was randomly downsampled by half, simulating a scenario where non-Swiss countries only sequenced half as much. A, B: Range and mean of the number of imports estimated with the phylogenetic analysis. C, D: Number of laboratory-confirmed SARS-CoV-2 cases per day (gray). The blue and purple area show the range of the simulated number of VoC cases using imports from the ten reruns. E, F: The area represents the range of the proportion of reported Alpha and Delta among all SARS-CoV-2 infections, whereas the line represents our estimates from the baseline (see Figure 4). Gray: Genomic surveillance data with a 95% confidence interval (CI). Blue: Liberal approach. Purple: Conservative approach.

In general, we found that using fewer context sequences only slightly reduced the number of imports estimated with the liberal approach to 975 (range: 954-995) and 1,064 (range: 1,046-1,078) compared to 1,038 and 1,347 imports of Alpha and Delta into Switzerland, respectively. For the conservative approach we also estimated slightly fewer imports, namely 331 (range: 313-360) and 362 (range: 342-380) compared to 383 and 455 imports of Alpha and Delta into Switzerland, respectively. These findings indicate that the actual number of imports is likely to be between the liberal and conservative estimates but also indicates that the estimates are not deeply dependent on high sequencing coverage around the world.


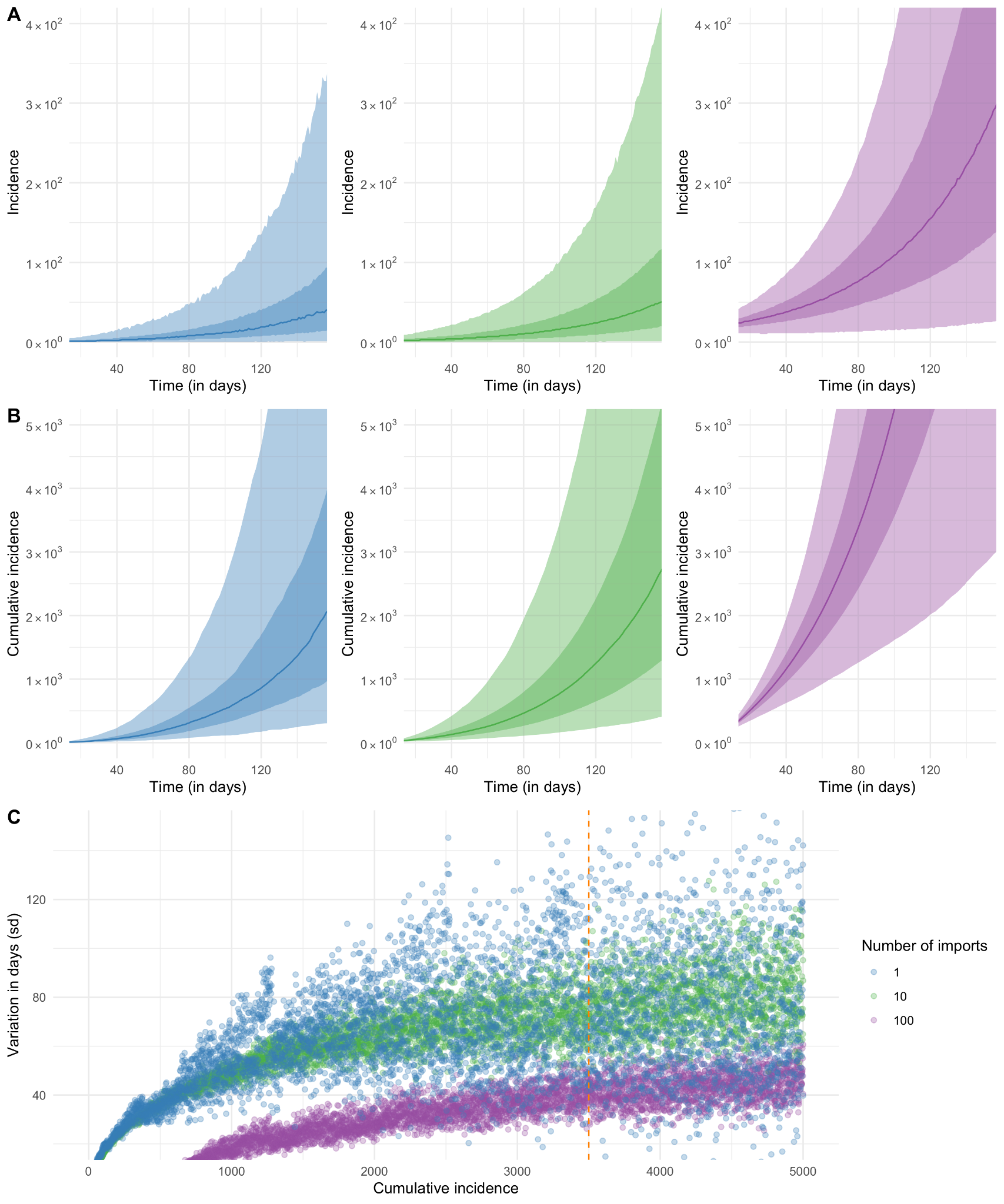


**Fig I.** Stochastic effects during the early growth phase of SARS-CoV-2 variants. A, B: Time to reach a certain (cumulative) incidence. Shaded regions correspond to the 50% and 95% interval of all simulations. C: Variation in the time to reach a certain cumulative incidence expressed as standard deviation.

**Reference**

1. Hodcroft EB, Zuber M, Nadeau S, Vaughan TG, Crawford KHD, Althaus CL, et al. Spread of a SARS-CoV-2 variant through Europe in the summer of 2020. Nature. 2021 Jul 29;595(7869):707–12.
2. du Plessis L, McCrone JT, Zarebski AE, Hill V, Ruis C, Gutierrez B, et al. Establishment and lineage dynamics of the SARS-CoV-2 epidemic in the UK. Science. 2021 Feb 12;371(6530):708–12.
