# Supplementary material for: Importation of Alpha and Delta variants during the SARS-CoV-2 epidemic in Switzerland: Phylogenetic analysis and intervention scenarios
Source: PLoS Pathog. 2023 Aug 10;19(8):e1011553. doi: 10.1371/journal.ppat.1011553 (PMC10443857; doi:10.1371/journal.ppat.1011553)
Supplement: S1 Table — (DOCX) [file ppat.1011553.s002.docx]

**Supplementary Table**

**Sup Table 1.** Estimated importation of VoCs and simulated impact on the SARS-CoV-2 epidemic in Switzerland. Abbreviation: VoC, variant of concern.

|  | **Alpha** | | **Delta** | |
| --- | --- | --- | --- | --- |
|  | **Liberal** | **Conservative** | **Liberal** | **Conservative** |
| **Estimated imports from the phylogeny** | 1,038 | 383 | 1,347 | 455 |
| **Simulation period** | 1 Oct 2020 - 1 May 2021 | | 1 Feb 2021 - 1 Sept 2021 | |
| **Total simulated reported cases** | 593,418 | 592,768 | 288,397 | 271,702 |
| **Number of simulated variant cases** | 97,116 (16%) | 70,898 (12%) | 110,596 (38%) | 87,861 (32%) |
| **Date by which 50% of cases are the variant (dominance)** | 05 Mar 2021 | 22 Mar 2021 | 30 Jun 2021 | 09 Jul 2021 |
