# Supplementary material for: Importation of Alpha and Delta variants during the SARS-CoV-2 epidemic in Switzerland: Phylogenetic analysis and intervention scenarios
Source: PLoS Pathog. 2023 Aug 10;19(8):e1011553. doi: 10.1371/journal.ppat.1011553 (PMC10443857; doi:10.1371/journal.ppat.1011553)
Supplement: S2 Table — (PDF) [file ppat.1011553.s003.pdf]

SUPPLEMENTAL TABLE

Data Availability

GISAID Identifier: EPI_SET_221003xn
doi: 10.55876/gis8.221003xn

All genome sequences and associated metadata in this dataset are published in GISAID's EpiCoV database. To view the contributors of each individual sequence with details such as accession number, Virus name, Collection date, Originating Lab and Submitting Lab and the list of Authors, visit 10.55876/gis8.221003xn

Data Snapshot

- EPI_SET_221003xn is composed of 32,438 individual genome sequences.
- The collection dates range from 2019-12-24 to 2021-07-31;
- Data were collected in 130 countries and territories;
- All sequences in this dataset are compared relative to hCoV-19/Wuhan/WIV04/2019 (WIV04), the official reference sequence employed by GISAID (EPI_ISL_402124). Learn more at https://gisaid.org/WIV04.
