# Supplementary material for: Importation of Alpha and Delta variants during the SARS-CoV-2 epidemic in Switzerland: Phylogenetic analysis and intervention scenarios
Source: PLoS Pathog. 2023 Aug 10;19(8):e1011553. doi: 10.1371/journal.ppat.1011553 (PMC10443857; doi:10.1371/journal.ppat.1011553)
Supplement: Attachment — Submitted filename: Response Imports of variants.pdf [file ppat.1011553.s004.pdf]

Point-by-point-response

Dear Prof. Swanstrom,

We are highly grateful for the opportunity to resubmit our manuscript entitled "Importation of Alpha and Delta variants during the SARS-CoV-2 epidemic in Switzerland: phylogenetic analysis and intervention scenarios". During our revision, we were excited to see the publication of the article by Dellicour et al. (2023, PLOS Pathogens) who describe the dispersal dynamics of SARS-COV-2 lineages in New York City, underlining the importance of phylogenetic analyses to track the importation and subsequent spread of variants. We highly appreciate your feedback and that of the reviewers. We believe their suggestions have greatly improved our manuscript. Below we provide a point-by-point response to the reviewers' comments. Changes in the manuscript are highlighted in green.

Reviewer #1: Dr. Bettisworth

> *Part I - Summary*
> *This paper attempts to combine compartmental models of epidemiology and transmission graphs derived from phylogenetic analysis to test various policy counterfactuals and their effects. In general, the paper is vague on details regarding the phylogenetic analysis, and therefore fails to be a convincing argument. My specific issues are detailed below, but in short the authors took an inferred phylogenetic tree as ground truth to perform their analyses. While this is a normal assumption in many other papers, it is particularly problematic when applied to SARS-CoV-2 data. Therefore, I request that the authors improve the robustness of their analysis by considering phylogenetic uncertainty (some suggestions have been listed below).*
>
> *I cannot speak to the portion of analysis based on compartmental models, however it seems to my eyes to also be quite vague on details.*
>
> *At the moment, there is nothing to my eyes that would constitute unfixable errors in methodology. Furthermore, the testing of policy interventions is self-evidently valuable, regardless of the particular conclusions. However, the amount of work required to make those conclusions robust and reliable might be significant, as the authors made assumptions about their data (that phylogenetic analysis is reliable in this case) which are not born out by prior work.*

We thank the reviewer for all comments and especially for all technical inputs regarding the phylogenetic analysis. We included a sensitivity analysis to test the robustness of our results, extended the method section in the main text, and added more details to the supplement to make the analysis more clear and enable reproducibility. A detailed response to the issues raised by the reviewer is given below.

> *Part II – Major Issues: Key Experiments Required for Acceptance*
> *The citations in the PDF provided are essentially dead links. If clicked on, the link directs the user to a Zotero page with no meaningful information. This should be corrected before the final publication, and it makes reading the paper and checking the references a bit difficult.*

We apologize for the links, they were automatically generated by Zotero and we didn't realize they did not lead anywhere. We removed these links in the revised version.

> *The description of the conservative and liberal approaches to count imports is quite vague. For the conservative estimate, it isn't clear what "counts" as a subtree. The Figure 1A suggests that it is some maximal clade, which contains all the Swiss sequences. However, if this is the case, then by*

> When Swiss and non-Swiss sequences intermix within a subtree, the conservative approach count[s] this as only one import.

the conservative estimate would always be 1, for any tree. However, Figure 4A and B show conservative counts much higher than 1, so there must be some method to picking the subtrees other than "maximal clades" which is not described in the paper.

The liberal approach seems to be more clear, as it seems to be counting the number of "monophyletic" clades in the tree, and probably includes individual tips. In this case, it seems it would be the minimum number of clades s.t. all clades are "monophyletic" and all Swiss sequences are accounted for. Of course, this definition can be made more rigorous by describing the clade as sets or partitions on tips. Nonetheless, as this metric is an important result of the paper, a explicit and rigorous definition should be presented.

Secondly, the above definitions are in conflict with the first sentence:

> We inferred an import whenever a node without Swiss sequences led to a node with Swiss sequences.

Which is
a) very vague as there is no description of how internal nodes are assigned to be either Swiss or non-Swiss and
b) contradictory with later definitions.
Basically, the section describing this section needs to be made much more clear.

Thank you for highlighting that the methodology we used was not described appropriately. We added extensive supplementary material to better explain how the collapsing works. Essentially, because we were not interested in any diversification of sequences once they were spreading in Switzerland, we collapsed clusters of Swiss-only clusters intwards (towards the root) until they become part of a polytomy that also contains non-Swiss clusters (a putative introduction). We improved the explanation of our liberal and conservative approach in the method section, as follows (p. 3, lines 36-52, p.4, lines 1-3):

“As previously described in Hodcroft et al. (3), we collapsed subtrees that contain only sequences from a single country into the parental node recursively (see Supplementary Methods for more detailed explanation). Because both Alpha and Delta originated outside of Switzerland, the roots of these variants are non-Swiss, and thus, we inferred a putative import whenever a node without Swiss sequences led to a node with Swiss sequences. We used two approaches to estimate the number of imports, referred to as the liberal and conservative phylogenetic approach, which infer upper and lower bounds on the number of imports. The liberal approach considered any mixed Swiss and non-Swiss node as an introduction (Fig 1A). On the other hand, when Swiss and non-Swiss sequences formed a subtree (with mixed-country nodes leading to more mixed-country nodes), the conservative approach counted all directly linked mixed-country nodes as only one import, with further non-Swiss sequences assumed to originate from parallel evolution outside of Switzerland or exports from Switzerland (Fig 1B).

The liberal approach may overestimate imports, as further exports from Switzerland to other countries or diversification within Switzerland could be wrongly considered imports. In contrast, the conservative approach considered only the earliest clusters with a Swiss sequence as imports, which means that further potential imports were strictly excluded. We chose the date of the earliest sequence of a cluster as the import date and shifted the date backwards by seven days to account for the delay from infection to case reporting, i.e. lag of detection. In a sensitivity analysis we used a range of three to thirteen days for the lag of detection derived from du Plessis et al. (2021) (6) (Sup Fig 1).”

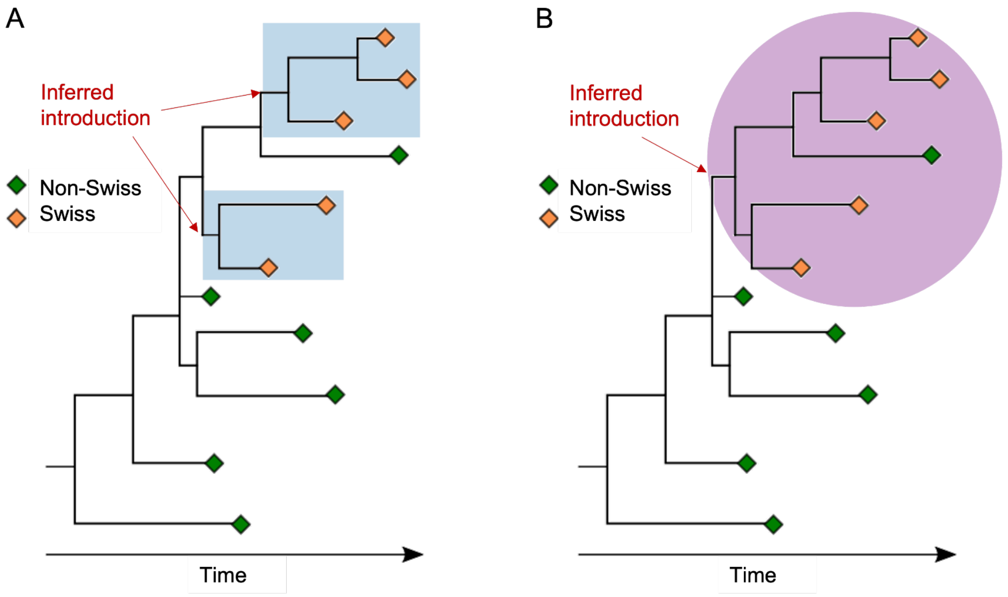

Fig 1: Illustrative example of the conservative and liberal approach to estimate the importation of SARS-CoV-2 variants of concern (VoC) to Switzerland. A: In the liberal approach, each subsequent subtree of only Swiss sequences is considered as a separate importation event. In this example, there would be two imports to Switzerland. B: In the conservative approach, subtrees with mixed Swiss and non-Swiss sequences are considered as one importation. Non-Swiss sequences are assumed to be exports from Switzerland or to originate from parallel evolution outside of Switzerland. In this example, there would be one import into Switzerland. More details are provided in the Supplementary Methods.

However, regardless of the particular definition of conservative and liberal estimates of imports, performing this operation on a particular tree is methodologically questionable. Recall that phylogenetic analysis of SARS-CoV-2 data is difficult[1], and the phylogenies are unstable [2]. Sequences which are sampled close together often differ by only a sites, leading to the case where the support for any _particular_ phylogeny is not very strong. Furthermore, sequencing error from some labs might bias the results in this case [3]. The phylogeny in this paper appears to be constructed via the Nextstrain pipeline [4], which is well regarded. However, this pipeline doesn't escape the fundamental problems that there doesn't seem to be enough signal to reliably resolve the phylogeny to the detail required for this paper.

This difficulty in resolving the fine grained phylogenetic relationships between sequences is a particular issue for this paper, as a core metric relies on accurately and reliably being able to resolve these relationships. I hesitate to give a specific instruction to fix methodology, but in this case _some_ level of uncertainty analysis must be done. The authors could construct a tree set like in [1], or to sample trees from the posterior in some inference program (such as BEAST or RevBayes). In either case, the authors should rerun their analyses on these sample trees, in order to ensure that the results are stable. It is possible that the conclusions of this paper hold, however it is not clear at this point that the conclusions of this paper are dependant on a particular (and possibly incorrect) tree.

[1]: https://doi.org/10.1093/molbev/msaa314
[2]: https://doi.org/10.1371/journal.pgen.1009175
[3]: https://virological.org/t/issues-with-sars-cov-2-sequencing-data/473
[4]: Though this is never explicitly stated, and can only be inferred via a reference to another paper. However, that paper has procedures for sub sampling the dataset which do not directly translate to the subject matter at hand. In particular, the number of sequences reported to be

in the tree in Hodcraft 2021 is different than then number of sequences reported in this paper.

We thank the reviewer for bringing up the important point of phylogenetic uncertainty. As covered in the papers cited by the reviewer, particularly at the beginning of the pandemic, phylogenetic reconstruction was a challenge, with low genetic diversity, unchecked initial spread of SARS-CoV-2, and low sequencing coverage, resulting in large polytomies that could not be resolved. However, by the advent of Alpha sequencing coverage was higher, international spread was somewhat constrained through awareness, tests, & travel restrictions, and genetic diversity grew as new variants evolved. In many cases (and likely uniquely for SARS-CoV-2), we had samples for each mutation as they appeared, giving us confidence in accurate reconstruction. Still, the presence of large polytomies at the roots of novel variants is common before more genetic diversity appears and can make the resolution of precise phylogeographic inference challenging. In our analyses we did not attempt to resolve these polytomies and instead always conservatively considered them as one introduction. Similarly, as we were only interested in introductions, we collapsed much of the detailed tree structure and looked only for transitions from non-Swiss to Swiss sequences as signatures of introductions. We added a new ‘Supplementary Methods’ to explain this in more detail. We also improved how this is explained in the text, below.

However, we also welcome the suggestion to find a way to include multiple runs to assess the impact of phylogenetic uncertainty in our estimates. The size of our datasets would make running a BEAST analysis computationally difficult, but we now included 10 additional reruns of our phylogenetic inference (using IQTREE as implemented in Nextstrain’s augur) for our Alpha and Delta scenarios. In addition, our analysis is most likely impacted by how well we can detect introductions from the Swiss sequences available (if an introduction or its descendants is not sequenced in Switzerland, we will miss it) and the global sequences available (separate introductions to Switzerland could be considered one introduction if there is no sequence to ‘split’ the Swiss introductions apart). Though we cannot include more Swiss sequences (we already use all available that pass quality criteria), we can include fewer global sequences to simulate overall less sequencing in the rest of the world and see how this impacts our analysis. Thus, we downsample the available global sequences to half before choosing those to include in our analyses and rerunning the phylogenetic analysis for each of our ten reruns. We added this sensitivity analysis in the method and supplementary material (Sup Fig 5).

We included further details of our phylogenetic method in the methods and discussion, as we showed in the previous response and adapted our method as follows (p. 3, lines 8-32):

“We used a combination of code from CoVariants.org and in-house scripts (available at https://github.com/emmahodcroft/Intros-CH-AlphaDelta) to select all Alpha (Nextstrain clade 20I) and Delta (Nextstrain clades 21A, 21I, and 21J) sequences sampled in Switzerland prior to 31 March 2021 and 31 July 2021, respectively, which are the dates when the proportion of each VoC was over 90% among all sequenced cases in Switzerland. We then ran the selected sequences through the Nextstrain ncov pipeline (35), as modified for CoVariants.org, to generate ‘focal’ phylogenies (available at https://github.com/emmahodcroft/ncov_2021/tree/random_context_reduce). We minimized bias and improved phylogenetic inference by masking highly homoplastic sites and removing sequences with fewer than 27,000 bases, as recommended by de Maio et al. (2020) (36). These focal trees contained all Alpha or Delta sequences from Switzerland before the 90% VoC representation date which passed quality control criteria. Alongside these, to maximize the chance of including closely-related non-Swiss sequences, which allows detection of a putative importation, we included up to 10,000 non-Swiss, global ‘context’ sequences that were most genetically similar to our focal set. We chose the number of genetically similar sequences in order to have a dataset of approximately 15,000 to 18,000 sequences for computational tractability, while resulting in 1.25 to 2 genetically similar global sequences per focal sequence. In addition, approximately 200 random ‘background’ sequences, distributed through time, were included to ensure the tree rooted correctly.

Both of these context and background sequence sets were generated using the algorithms in the Nextstrain ncov pipeline (see Supplementary Methods), and were also sampled prior to 31 March 2021 and 31 July 2021 for Alpha and Delta respectively. The final set of sequences that we used for the analysis can be seen on GISAID under EPI_SET_221003xn; 93% of the Swiss sequences are also available openly (Sup Table 2). As a sensitivity analysis, we randomly downsampled all available sequences prior to our cutoff dates by 50% before selecting the most 10,000 genetically similar sequences, and repeated this ten times. The objective was to assess the influence of context sequencing coverage on our phylogenetic analysis and estimates of the imports."

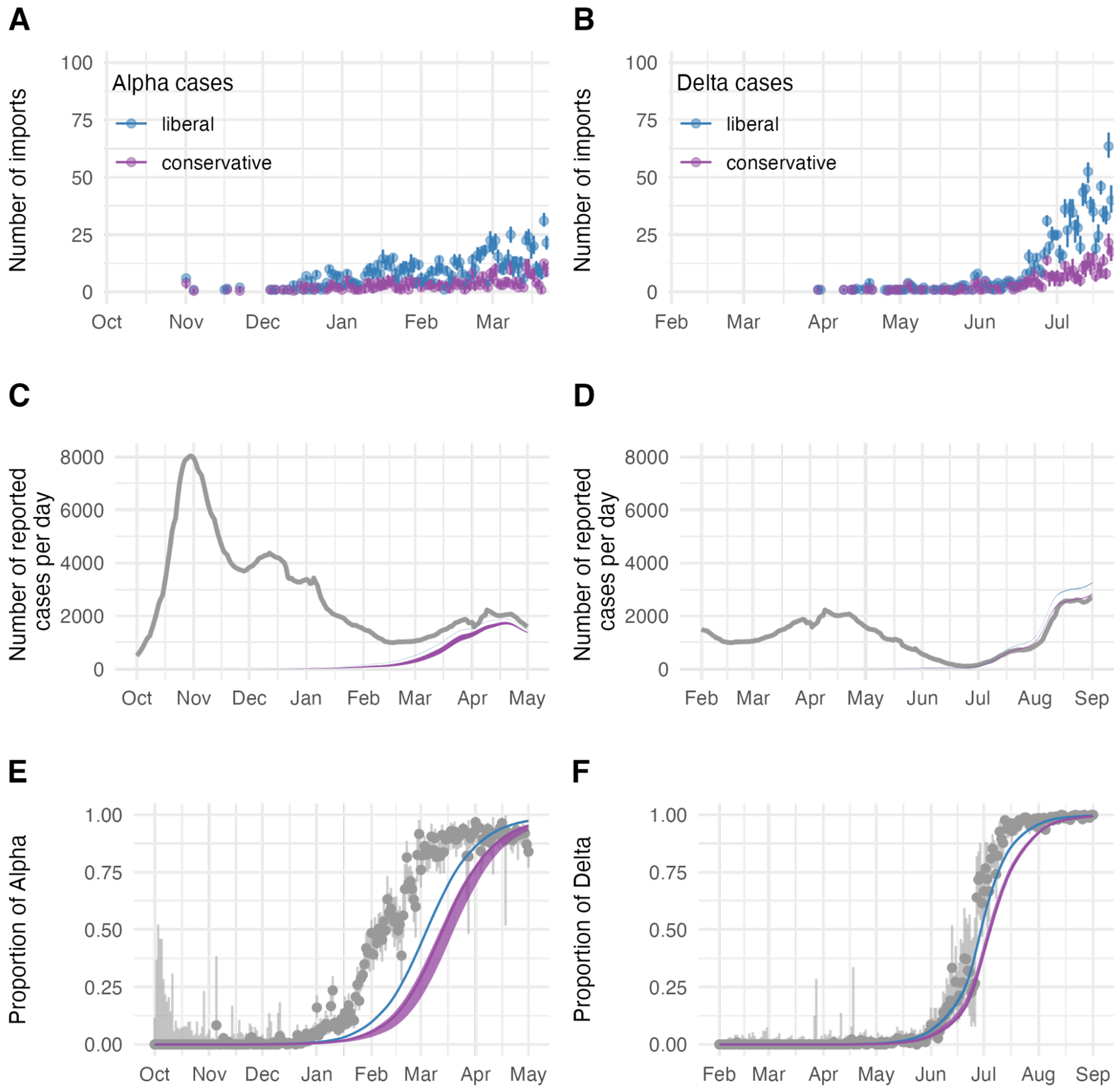

Sup Fig 5: Dynamics of Alpha and Delta importation to Switzerland. Range of the estimated number of imports and resulting model for ten reruns of the Alpha and Delta analysis, where the number of possible context sequences was randomly downsampled by half, simulating a scenario where non-Swiss countries only sequenced half as much. A, B: Range and mean of the number of imports estimated with the phylogenetic analysis. C, D: Number of laboratory-confirmed SARS-CoV-2 cases per day (gray). The blue and purple area show the range of the simulated number of VoC cases using imports from the ten reruns. E, F: The area represents the range of the proportion of reported Alpha and Delta among all SARS-CoV-2 infections, whereas the line represents our

estimates from the baseline (see Figure 4). Gray: Genomic surveillance data with a 95% confidence interval (CI). Blue: Liberal approach. Purple: Conservative approach.

The Supplementary Figure 3 is followed by corresponding Supplementary Text: “In general, we found that using fewer context sequences only slightly reduced the number of imports estimated with the liberal approach to 975 (range: 954-995) and 1,064 (range: 1,046-1,078) compared to 1,038 and 1,347 imports of Alpha and Delta into Switzerland, respectively. For the conservative approach we also estimated slightly fewer imports, namely 331 (range: 313-360) and 362 (range: 342-380) compared to 383 and 455 imports of Alpha and Delta into Switzerland, respectively. These findings indicate that the actual number of imports is likely to be between the liberal and conservative estimates but also indicates that the estimates are not deeply dependent on high sequencing coverage around the world.“

We also discuss suggested references as follows (p. 11, lines 31-41): “Finally, the phylogenetic analysis has intrinsic limitations due to the fact that not every infection is detected or sequenced, and interpretation during outbreaks requires caution (2,49), which was also highlighted at the beginning of the pandemic by Morel et al. 2020 (50). This is specifically true for Alpha, which appeared at the end of 2020, prior to sequencing in Switzerland being scaled up considerably during 2021. To further mitigate the impact of sequencing error and unreliable sequences, as highlighted by Turakhia et al. (2020) (51), we excluded sequences with poor coverage and low quality control scores and masked homoplastic sites. There is currently no gold standard for estimating imports. Uncertainty in creating and interpreting phylogenies can lead both to imports being underestimated due to considering only one importation event per cluster and overestimated due to within-country diversification or further exports. In our analysis we aimed to address this issue, by using two approaches to estimate lower and upper bounds on the number of imports.“

> *Part III – Minor Issues: Editorial and Data Presentation Modifications*
> *Honestly, this section is quite short. I stopped adding to it after I realized that significant changes to the methodology would have to be made.*

We hope that we appropriately dealt with the reviewer’s comments by extending the methods section and adding additional supplementary material as described above.

> Page 4, starting on 116:
> > When Swiss and non-Swiss sequences intermix within a subtree, the conservative approach counted this as only one
> > import, with further non-Swiss sequences assumed to originate from parallel evolution outside of Switzerland or
> > exports from Switzerland (Fig 1A).
>
> The language here is a bit awkward, and I think there are some error in tenses. "Intermix" is present tense, but "counted" is past tense, and they should be the same tense, probably present tense.
>
> Page 4, starting on 119: Importation -> import
>
> Page 7, line 177: Importation -> import or "simulated the importation" -> "simulated importation"

We adapted our methods section for clarity, as outlined in our previous response. Notably, we opted for the term "import" where it is more appropriate than "importation," and we used the phrase "simulated importation" in p. 5, lines 35-36: “We simulated importation of Alpha and Delta to Switzerland during the period from 1 October 2020 to 1 May 2021 and 1 February 2021 to 1 September.”

Reviewer #2: Prof. Alizon

Part I - Summary
The authors combine phylogenetic inference and mathematical models to explore the impact of border closure to prevent the spread of SARS-CoV-2 variants in Switzerland. The phylogenetic inference was performed on sequences from the Alpha and Delta variant. By inferring ancestral states, the authors could estimate a conservative and a liberal number of introductions. They then used these estimates to parameterise an SEIR transmission model with two viral strains (a resident and a mutant) and performed counterfactual scenarios to explore the impact on the time until variant dominance of closing border for a given duration or increasing surveillance.

I found the phylogenetic inference impressive and the mathematical modelling robust. The combination of the two is promising and, generally, the authors are also very aware of the limitations of their approach. However, I still have a few concerns or questions. The main suggestion is to better demonstrate the added value of combining phylogenetic inference and compartmental modelling, for instance by performing sensitvity analyses but also by better discussing earlier studies on similar topics.

Thank you very much for your positive feedback and raising some important areas where our manuscript can benefit from greater clarity and additional discussion.

Part II – Major Issues: Key Experiments Required for Acceptance
1) Sampling rate
The authors are working with an absolute number of introductions in the country. Intuitively, this number seems very dependent on the sampling rate (proportion of the infections sequenced), although potentially not in a linear way. Furthermore, as the authors accurately acknowledge in the Discussion, the nature of the dataset used as a reference may also affect the results. On both these topics, the sensitivity analyses performed seem a bit light (or absent).

First, the authors could subsample their dataset to see how the number of introductions scales with the subsampling. I think this can be done easily because it does not require to re-infer the phylogeny. Second, the authors could try a few different reference datasets to show that their estimates remain unaffected. For the latter, the computational burden is higher so perhaps a few datasets will suffice.

We appreciate your suggestions regarding the potential impact of the sampling strategy on our findings, and agree that sampling/sequencing rates could impact how we can detect importations. In response to this concern, we conducted a sensitivity analysis to quantify the variations of estimated imports resulting from resampling context sequences (see also our response to reviewer #1's second point). In 10 replicates for each variant, we downsampled all available sequences by 50% before selecting the 10,000 most genetically similar sequences to our Swiss sequences to include in our phylogenies. This simulates a scenario where fewer sequences were available from the rest of the world to help us distinguish imports. It would also be possible to include more than 10,000 context sequences, but this would become computationally difficult. We also considered downsampling the Swiss sequences, but as this would only result in potentially removing introductions and/or moving them later in time, this would likely lead to a poorer fit to the real data.

Detailed information regarding this analysis has been incorporated into both the method and supplement accordingly (p. 3, lines 29-32): "As a sensitivity analysis, we randomly downsampled all available sequences prior to our cutoff dates by 50% before selecting the most 10,000 genetically similar sequences, and repeated this ten times. The objective was to assess the influence of context

sequencing coverage on our phylogenetic analysis and estimates of the imports."

And in our Supplement as follows: "In general, we found that using fewer context sequences only slightly reduced the number of imports estimated with the liberal approach to 975 (range: 954-995) and 1,064 (range: 1,046-1,078) compared to 1,038 and 1,347 imports of Alpha and Delta into Switzerland, respectively. For the conservative approach we also estimated slightly fewer imports, namely 331 (range: 313-360) and 362 (range: 342-380) compared to 383 and 455 imports of Alpha and Delta into Switzerland, respectively. These findings indicate that the actual number of imports is likely to be between the liberal and conservative estimates but also indicates that the estimates are not deeply dependent on high sequencing coverage around the world."

We added this interpretation to the result section as follows (p. 8, lines 12-14): "In our sensitivity analysis where we removed half of the available non-Swiss sequences before identifying the most genetically similar sequences to our Swiss sequences, the liberal and conservative approach had similar results as using the baseline sampling method (Sup Fig 5)."

Finally, we adapted our discussion as well (p. 10, lines 21-24): "The actual number of imports is likely between these estimates. From our sensitivity analysis, we saw that even if there had been substantially less sequencing outside of Switzerland to help infer imports, we would still detect around the same number of introductions."

> *2) Model results*
> *One of the main results is that the model underestimates the increase in variant frequency, even when using the liberal definition of importation events. This lag is less pronounced for Delta than for Alpha. In my opinion, this can either come from the importation rate or from some of the model specifications. At any rate, the origin of this mismatch and why it is more pronounced for the Alpha variant should be explored more thoroughly. One possibility could be to use the observed data to infer the VOC import rate instead of fixing it (unless another parameter seems less reliable).*

We thank the reviewer for pointing out that we could explain the observed discrepancy more thoroughly. Indeed, there is a difference between our findings for Alpha compared to Delta, i.e., the estimates for Alpha lagged more than Delta. The lag of dominance can be reduced by increasing the lag of the detection of importation; du Plessis et al. (2021) suggested this can be 8.22 ± 5.21 days. We used 7 days, but agree it is interesting to explore the effect of different lag times. If we use the range from 3 to 13 days (incorporating the range estimated by du Plessis et al. (2021)) the curve has a broader range (Sup Fig 1).

The difference in the modeled dynamic of Alpha and Delta could also be due to sequencing coverage, e.g., for Alpha in December 2020 the sequencing coverage in Switzerland was 2% where it was 10% for Delta in April 2021 (see *our world in data*), which could mean that we missed detecting some early imports. Moreover, for Alpha, the early clusters were larger than for Delta, which might indicate that due to low sequencing coverage in Switzerland but also overall, we were less likely to split clusters into separate introductions (Sup Fig 4). These reasons (coverage, lag time, background sampling, missing of early imports) can influence the accurate capture of (dominance) dynamics of VoCs. In particular, early introductions are likely critical to when a VoC begins spreading (and thus reaches dominance), but were also probably easiest to miss in the case of Alpha, due to lack of awareness of VoCs (as Alpha was the first) and low sequencing coverage.

We address this in our method and supplement (p. 3, lines 52, p.4, lines 1-3): "We chose the date of the earliest sequence of a cluster as the import date and shifted the date backwards by seven days to account for the delay from infection to case reporting, i.e. lag of detection. In a sensitivity analysis we used a range of three to thirteen days for the lag of detection derived from du Plessis et al. (2021) (6) (Sup Fig 1)."

We address this in our results (p. 7, lines 28-30): "The simulated timing of dominance lagged estimates from sample data more for Alpha than for Delta, which might have been influenced by missing early imports indicated by larger clusters for Alpha than for Delta at the beginning (Sup Fig 4)."

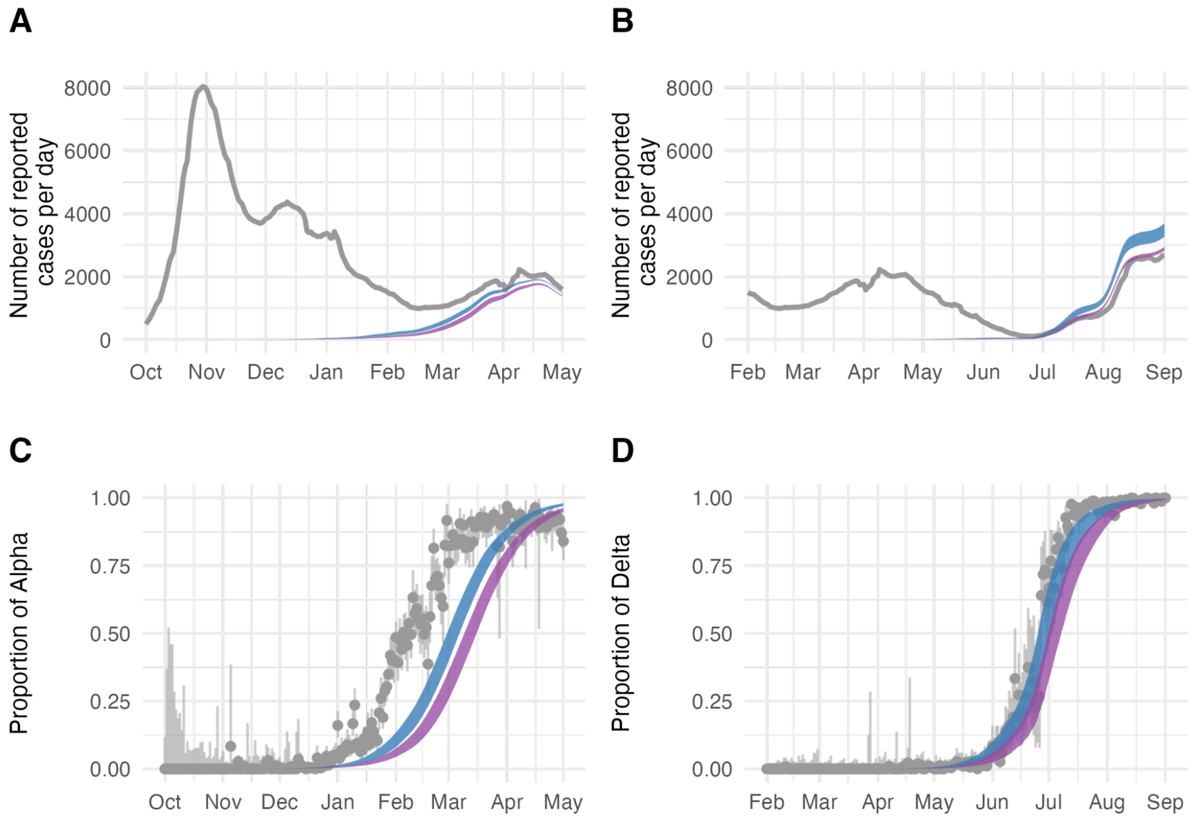

Sup Fig 1: Dynamics of Alpha and Delta importation to Switzerland. This figure is similar to Figure 4, but the range was received from eleven simulations using different times of detection lags, namely 3-13 days in 1 day steps. This range was derived from du Plessis et al. (2021). A, B: Number of laboratory-confirmed SARS-CoV-2 cases per day (gray). The blue and purple area show the range of simulated number of VoC cases. C, D: The range of proportion of reported Alpha and Delta among all SARS-CoV-2 infections. Gray: Genomic surveillance data. Blue: Liberal approach. Purple: Conservative approach.

We address this issue in our discussion (p. 11, lines 23-32): "For example in Switzerland the sequencing coverage increased from 2% in December 2020 (just after the first importation of Alpha) to 10% in April 2021 (during the first importation of Delta) (47,48), and was on average 14% for our study period (1 October 2020 to 1 September 2021). The limited sequencing efforts during the introduction of the Alpha variant may hinder the detection of early imports and splitting clusters accurately. Sixth, the lag time of detecting an import and the true introduction might vary by testing and sequencing strategies, e.g., du Plessis et al. (2021) reported a detection lag of 3 to 13 days of the introduced cluster time of the most recent common ancestor (TMRCA) to the true importation (6). Finally, the phylogenetic analysis has intrinsic limitations due to the fact that not every infection is detected or sequenced, and interpretation during outbreaks requires caution (2,49)."

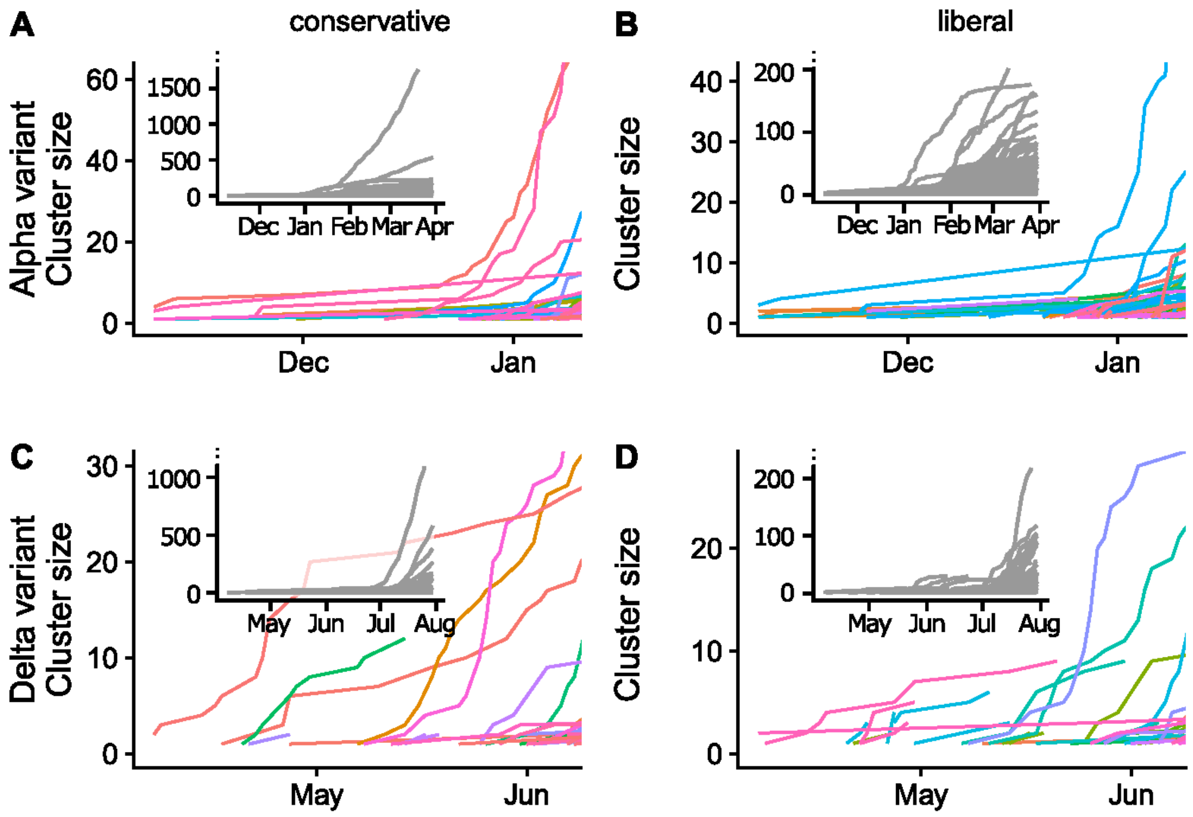

Sup Fig 4: The growth in Swiss sequences of the cluster that led to an import of a VoC. The plots show each cluster for the first two months as a line. The small zoomed plot shows the clusters over a longer time period. A: Alpha variant and conservative approach. B: Alpha variant and liberal approach. C: Delta variant and he conservative approach. D: Delta variant and liberal approach.

> *3) Model parameterisation*
> *As earlier studies, e.g. Du Plessis et al (Ref. 6 in the manuscript, which could perhaps be discussed some more), the authors can infer the number but also the date of VOC introduction. I wonder if turning this into a constant input rate is not throwing some of the information away. Put differently, if the goal is to infer such a rate while all the other model parameters are known (see Table 1), then why not use a more appropriate dataset and, for instance, try to fit the proportion curves?*

We thank the reviewer for highlighting the study by du Plessis et al. (2021). We have now discussed their work in comparison with ours in more detail in our discussion (see above). However, we did not use a constant importation rate in our transmission model. ω denotes a time-dependent importation rate that is parameterized by the number of estimated imports from the phylogenetic analysis. We now better describe this feature of the model in our methods as follows (p. 5, lines 22-24): "ω_t corresponds to a time-dependent rate of importation of VoCs that was based on the daily number of estimated imports from the phylogenetic analysis (Fig 4AB)." We adapted Figure 2 and the text by changing ω to ω_t and Figure 4AB visualizes the days of the introduction of VoCs.

> *3) Insights*
> *Overall, I found that the discussion about the insights from the study was somehow limited. The most interesting bit was the second to last paragraph from line 361 about Omicron. One way to improve the discussion could be to refer to earlier studies. For instance, a quick search online pointed towards a paper from Scalia Tomba & Wallinga (2008, Math Biosci) with an eloquent title: "A simple explanation for the low impact of border control as a countermeasure to the spread of an infectious disease". More recently, Pung et al (2023, BMC Med) seem to have developed a more detailed model addressing similar questions.*

Related to this point, showing the added value of fitting the number of introductions from the sequence data rather than on the relative variant proportion might help. For this, discussing differences with other studies that performed similar inferences, e.g. Du Plessis et al in England, seems important.

We thank the reviewer for pointing out that we could more thoroughly expand our discussion. As we mentioned in our response to point 2, we do estimate the number of introductions from the sequence data rather than using a constant rate or relying on the variant proportions.

From the useful suggestions and papers, we have incorporated the following additions (p. 10, lines 32-33): "Tomba and Wallinga (2008) also showed that with 90% or greater reduction in imports the time to dominance can be delayed by more than a week (44)."

p. 11, lines 50-52, p. 12, lines 1-15: "Tomba and Wallinga (2008) emphasized the significance of estimating the impact of travel on infectious disease transmission and the potential effectiveness of interventions on the local spread, particularly highlighting that initial importation events will eventually lead to a local epidemic if not contained, whereas importations after local expansion has begun are less impactful (44). Based on routine surveillance data from FOPH, we previously extrapolated a total of 6,211 and 37,061 imported cases in Switzerland during summer 2020 and 2021, respectively. Other studies also using phylogenetics estimated the number of imported infections during the early phase of the SARS-CoV-2 pandemic. For example, 13 and 101 introductions were estimated to South Africa over two and eight months (26,52), 120 introductions were estimated in Boston over three months (23), and more than 1,000 introductions were estimated to the UK over three months (6). During summer 2020, we estimated 34 to 291 introductions of the SARS-CoV-2 variant EU1 to Switzerland over four months (3). Phylogenetics offers an advantage in estimating imports by leveraging sequence data that was readily produced by many countries in the pandemic, but can be limited by insufficient coverage to detect importations, fluctuations in coverage, and dependence on other countries to also sequence sufficiently. Methods such as that employed by Pung et al. in Singapore can incorporate detailed case finding and contact tracing data to break down the impact of different measures even further, but the availability of such data varies widely across countries and pandemic stage (53)."

4) Data sharing
Before criticising, I want to stress that the authors made impressive efforts to share the code they developed! Perhaps they could also consider a more permanent repository than GitHub (either PLoS Pathogens supplementary materials or a repository with a DOI).
However, given the current context about GISAID usage, it seems important to check that the laboratories who contributed essential data to this work (so the Swiss Alpha and Delta sequences) were contacted to be involved in the study or that there is a national agreement about genetic data usage (akin to the UK genomics consortium). In any case, the sentence in line 388 suggesting that the data is openly shared by GISAID is inaccurate: there are restrictions to this use.

We appreciate your comment raising an important point about data accessibility and credit for data generation. While Switzerland does not have a formal group that can be included as an author, we are actively participating in the Swiss genomic surveillance program and reached out to the former president of the Swiss National COVID-19 Science Task Force for guidance on how to attribute the sequences we used. On their advice, we have included the following statements to address this (p. 3, lines 6-8): "We accessed sequencing data via GISAID (33) on 15 February 2022, including data generated in Switzerland as part of a federal consortium (34), to ensure the considered sequences encompass the introduction periods of Alpha and Delta into Switzerland."

and in our Acknowledgment as follows: "We gratefully acknowledge and thank all labs around the

world that have collected and shared SARS-CoV-2 sequences we used in our study. A complete list of the labs that generated the data we used from GISAID can be found at doi.org/10.55876/gis8.221003xn. In particular, we want to especially thank the Swiss laboratories that perform SARS-CoV-2 sequencing, namely ‘Hôpitaux Universitaires Genève’ (HUG), the ‘Centre hospitalier universitaire vaudois’ (CHUV), the 'Universitätsspital Basel', the ‘Institut für Infektionskrankheiten’ (IFIK) of the University of Bern, ‘Institute of Medical Virology’ (IMV) of the University of Zurich, the ‘Ente Ospedaliero Cantonale’ (EOC) in Bellinzona, and ‘Institut Central des Hôpitaux du Valais’.”.

We agree that previously we had not fully accurately described the availability of the sequences, and agree that providing the GitHub repositories in a more permanent manner is helpful. We have amended our data availability statement as follows: “The data of confirmed SARS-CoV-2 cases are openly shared by the Swiss Federal Office of Public Health (FOPH) and sequence data are available on GISAID after registration, as EPI_SET_221003xn. Most Swiss sequences (93%) that we used are also available openly, see Supplementary Table 2 for list of accession numbers. Our code is openly accessible in the following repositories: github.com/ISPMBern/voc_imports_ch (https://doi.org/10.5281/zenodo.7994708) for the transmission modeling, https://github.com/emmahodcroft/ncov_2021/tree/random_context_reduce (https://doi.org/10.5281/zenodo.7970773) for the phylogenetic analysis and github.com/emmahodcroft/Intros-CH-AlphaDelta (https://doi.org/10.5281/zenodo.7970756) for the inference of importations.”

> *Part III – Minor Issues: Editorial and Data Presentation Modifications*
> *Line 48: the term "lockdown" should be handled with care because it has different meanings in different countries or contexts. Perhaps the authors should spend a bit more time defining it carefully to avoid potential misunderstandings.*

Thank you for highlighting that the term lockdown should be used carefully. We changed the corresponding section as follows (p. 2, lines 8-11): “It was previously shown that the resumption of travel in summer 2020 after strict control interventions that - while varying by country - often involved border closures, working from home and restricting social contacts, led to the importation of a new SARS-CoV-2 variant that fuelled epidemics in countries with low-incidence (2,3).”

- *line 157: I think kappa estimates a growth advantage rather than a transmission advantage (if a variant causes longer infections with a similar transmission rate it would also be captured). Perhaps update the formulation?*

We thank the reviewer for pointing out that the definition and calculation of κ requires further description. We extended the corresponding text in the Methods section as follows (p. 5, lines 3-14): “κ denotes the increased transmissibility of the imported VoC. We calculated κ from the estimated growth advantage ρ of the new VoC compared to the previously circulating variants using a logistic growth model (binomial regression) for the proportion of the new variant among all previously circulating variants (Sup Fig 2). Assuming no change in the generation time D and no immune evasion, $\kappa = \rho * D / R_w$ (11), where R_w is the effective reproduction number of the previously circulating variants during the time period of replacement. We sampled from that the publicly available estimates of the daily overall effective reproduction number R_e from 1 November 2020 to 31 January 2021 (early growth phase of Alpha) and from 1 April 2021 to 30 June 2021 (early growth phase of Delta), assuming the values correspond to R_w during these time periods (https://github.com/covid-19-Re) (Fig 3B) (28). Additionally, we sampled from the estimated ρ and calculated the median κ. We then expressed the time-dependent transmission rate as a function of the overall R_e as follows: (...)”

> *Table 1: I did not understand how the testing delay came into the model (to go from "tested" to "recovered").*

We agree that we could be clearer in explaining this. We updated the text to provide a more

comprehensive description of the tested and recovered compartment as follows (p. 5, lines 20-22): ”A fraction ε of infected infectious individuals enter a testing compartment where they get tested at rate ζ before entering the recovered compartment, whereas the remainder ($1-\varepsilon$) does not get tested and moves directly from the infected compartment to the recovered compartment.”

- *Page 9: It seems important to mention that the Alpha variant was first detected and studied because of the S-target gene failure. Sequencing confirmed the detection (showing that it was a variant) and was used later to study variant spread.*

Thank you for highlighting this point that detection and sequencing of the Alpha may have been facilitated due to the fact that the variant was detectable with routine molecular methods due to the S-target gene dropout. On the other hand, the sequencing coverage was higher during the emergence and spread of Delta. We added the following text to the Results section to discuss this point (p. 7, lines 21-24): “Notably, deletions in the Alpha variant meant that Polymerase Chain Reaction (PCR) testing methods targeting a particular section of the S gene failed, leading to ‘S gene target failure’ (SGTF), which allowed tracking of the spread of Alpha, and in some cases, preferential sequencing of Alpha cases (7).”

Figure 5: the nature of the x-axis is unclear (is it how long the border is closed?). Also, having all three panels on a line would help comparisons. Finally, I do not understand why panel c could not be a deterministic model as well.

We changed the labels of the x-axes to “Duration of closed borders (in days)” and “Duration of closed borders after awareness (in days)” and moved all three panels of the figure into one row. We changed the description of the scenario shown in panel C as follows (p. 6, lines 23-25): Increased surveillance of travelers: We assumed improved surveillance, quarantine, and isolation of travelers. We performed simulations where we reduced the number of simulated imports by sampling 1 to 99% of the estimated imports from the phylogenetic analysis.”

line 301: specify against which strain the transmission advantage is computed.

Please see our response to the reviewer’s earlier comment on the method and description to calculate the increased transmissibility κ.

Reference 37 (about computing variant growth advantage) is missing.

We apologize that the reference 37 was missing in ‘References’. We checked all references again and note that the reference order might have changed but you referred to following reference:

Chaoran Chen and others, CoV-Spectrum: analysis of globally shared SARS-CoV-2 data to identify and characterize new variants, *Bioinformatics*, Volume 38, Issue 6, March 2022, Pages 1735–1737, https://doi.org/10.1093/bioinformatics/btab856
