# Supplementary material for: Importation of Alpha and Delta variants during the SARS-CoV-2 epidemic in Switzerland: Phylogenetic analysis and intervention scenarios
Source: PLoS Pathog. 2023 Aug 10;19(8):e1011553. doi: 10.1371/journal.ppat.1011553 (PMC10443857; doi:10.1371/journal.ppat.1011553)
Supplement: Attachment — Submitted filename: Response Letter.pdf [file ppat.1011553.s005.pdf]

Point-by-point-response

Dear Prof. Shuo Su,

We are very grateful that the reviewers have carefully assessed our revision of the manuscript entitled "Importation of Alpha and Delta variants during the SARS-CoV-2 epidemic in Switzerland: phylogenetic analysis and intervention scenarios". We would like to thank both reviewers for highlighting some remaining issues to which we respond below. Changes in the manuscript are highlighted in green.

> **Part I - Summary**
> Reviewer #1: The authors have done an excellent job addressing the (possibly overly callous on my part) comments from the reviewers.
> At this time, I only have minor issues with the added explanation regarding the explanation of how imports are
> calculated.

We very much appreciated your earlier concerns that helped us to improve the manuscript.

> **Part II – Major Issues: Key Experiments Required for Acceptance**
> Reviewer #1: All of my major concerns have been addressed. I quite like the addition of the sensitivity analysis, and I think it
> greatly improves the reliability of the results presented in the paper.

We highly appreciate your feedback.

> **Part III – Minor Issues: Editorial and Data Presentation Modifications**
>
> Reviewer #1: The added explanation for how the number of imports is an improvement, but I still feel it is a bit unclear, especially
> after reading the supplement. However, I think that this can be fixed easily. On line 37:
>
> "we collapsed subtrees that contain only sequences from a single country into the parental node recursively" ->
> "we collapsed subtrees which contain only sequences from a single country into the parent node to form a polytomy. This process was repeated in a recursive 'bottom-up' fashion, such that every node eligible for collapse was collapsed."
>
> And add "we classified inner nodes based on the composition of their _direct_ children, after collapsing subtrees into polytomies" (or something like that, I don't mean to dictate your voice) to the same paragraph.

Thank you for your suggestions. We adapted the text as follows (p. 3-4, lines 40-52 and 1-2): "As previously described in Hodcroft et al. (3), we collapsed subtrees that contain only sequences from a single country into the parental node to form a polytomy. This process was repeated in a recursive 'bottom-up' fashion, such that every node eligible for collapsing was collapsed. After collapsing, we labeled internal nodes by the proportion of the geographic origin of their direct children (see Supplementary Methods for more detailed explanation). Because both Alpha and Delta originated outside of Switzerland, the roots of these variants are non-Swiss, and thus, we inferred a putative import whenever a node without Swiss sequences led to a node with Swiss sequences. We used two approaches to estimate the number of imports, referred to as the liberal and conservative phylogenetic approach, which infer upper and lower bounds on the number of imports. The liberal

approach considered any mixed Swiss and non-Swiss node as an introduction (Fig 1A). On the other hand, when Swiss and non-Swiss sequences formed a subtree (with mixed-country nodes leading to more mixed-country nodes), the conservative approach counted all directly linked mixed-country nodes as only one import, with further non-Swiss sequences assumed to originate from parallel evolution outside of Switzerland or exports from Switzerland (Fig 1B). Import events are recorded and were then used to parameterize the transmission model (see next section about the transmission model)."

Part I - Summary
Reviewer #2: I thank the authors for carefully answering my concerns. I found the new sensitivity analysis on the importance of sampling particularly thorough. The model specifications are also clearer and I realised that I misunderstood the definition of the import rate. Overall, I think the authors now better show what the phylogenetic component brings to these counterfactual models.

Thank you.

Part II – Major Issues: Key Experiments Required for Acceptance
Reviewer #2: My sole suggestion is still about this sampling rate because, if I think I now understand how it plays in the model, it might still not be the case. More precisely, in the Methods, \omega_t is never really properly defined. All that is said is that it "was based on the daily number of estimated imports" (page 5). Introducing it from the section "Phylogenetic analysis" (pages 3-4) would help insist on its added value. Furthermore, this time-varying property could also be underlined in the results because currently the authors only mention the total number of introductions ("we found 1,038 and 1,347 imports of Alpha and Delta into Switzerland, respectively", page 8). How did this input rate vary with time? Was it proportional to the circulation of the VOC in Europe or in the world? Addressing these questions by leaning on the (beautiful) Figure 4 would further show the importance of phylogenetic insights.

To avoid confusion between sampling of sequences and sampling of estimated imports, we changed the description of the third counterfactual scenario as follows (p. 7, lines 1-3): "Increased surveillance of travelers: We assumed improved surveillance, quarantine, and isolation of travelers. We performed simulations where we reduced the number of simulated imports by randomly selecting 1 to 99% of the estimated imports from the phylogenetic analysis."

Furthermore, we now better describe the parameterization of the time-dependent rate of importation ω_t in the Methods (p. 5, lines 27-30): "ω_t corresponds to the time-dependent rate of importation of VoCs. We parameterized the vector ω_t using the daily number of estimated imports from the phylogenetic analysis (Fig 4AB), e.g., ω_t for Alpha on 1 February 2021 was 8 and 2 for the liberal and conservative approach, respectively."

We also added the following sentence to the Method section - Phylogenetic analysis (p. 4, lines 1-2): "Import events are recorded and were then used to parameterize the transmission model (see next section about the transmission model)."

Part III – Minor Issues: Editorial and Data Presentation Modifications

Reviewer #2: page 7: The SGTF sentence might be better suited in the introduction but it's up to you.
page 10: Do you mean that Tomba and Wallinga (2008) showed that you need a 90% or greater reduction in imports so that the time to dominance can be delayed by more than a week?

p.2, lines 17-25: “Global genomic surveillance is vital to capture the transmission dynamics of different SARS-CoV-2 variants around the world. Genomic and epidemiological data allow characterization and quantification of the spread of VoCs (5–9). At the end of 2020, deletions in the Alpha variant meant that Polymerase Chain Reaction (PCR) testing methods targeting a particular section of the S gene failed, leading to ‘S gene target failure’ (SGTF), which allowed tracking of the spread of Alpha, and in some cases, preferential sequencing of Alpha cases (7). Alpha, that was initially detected in the UK, showing a 37%-100% increase in transmissibility over previously circulating variants (5,7–11). In early 2021, Alpha was continuously displaced by another VoC, Delta, which likely emerged in India (12).”

We now better describe the results from Tomba and Wallinga (2008) in the fourth paragraph of the discussion (p. 11, lines 45-50): “Tomba and Wallinga (2008) emphasized the significance of estimating the impact of travel on infectious disease transmission and the potential effectiveness of interventions on the local spread, particularly highlighting that initial importation events will eventually lead to a local epidemic if not contained, whereas importations after local expansion has begun are less impactful (51). Using an example of pandemic influenza, they estimated that a 90% reduction of imports would delay the first importation event by 11.5 days.”
